# Acupuncture or Acupressure at the Sanyinjiao (SP6) Acupoint for the Treatment of Primary Dysmenorrhea: A Meta-Analysis

**DOI:** 10.1155/2013/493038

**Published:** 2013-02-28

**Authors:** Ma-Na Chen, Li-Wei Chien, Chi-Feng Liu

**Affiliations:** ^1^Department of Nursing, Hsin Sheng College of Medical Care and Management, No. 418, Gaoping Section, Jhongfong Road, Longtan Township, Taoyuan Country 32544, Taiwan; ^2^Department of Obstetrics and Gynecology, Taipei Medical University Hospital, No. 252, Wu-Hsing Street, Taipei 11031, Taiwan; ^3^Department of Obstetrics and Gynecology, School of Medicine, College of Medicine, Taipei Medical University, No. 250, Wu-Hsing Street, Taipei 11031, Taiwan; ^4^Graduate Institute of Integration of Traditional Chinese Medicine with Western Nursing, National Taipei University of Nursing and Health Sciences, No. 365 Ming-Te Road, Beitou, Taipei 11211, Taiwan

## Abstract

This meta-analysis aimed to evaluate the effectiveness of acupuncture or acupressure at the Sanyinjiao (SP6) acupoint in relieving pain associated with primary dysmenorrhea. We searched the scientific literature databases to identify randomized controlled trials. The primary outcome was visual analogue scale (VAS) pain score. Three acupuncture and four acupressure trials were included in the meta-analyses. For the acupuncture analysis, there was no difference in the mean VAS score reduction between the SP6 acupoint and control (GB39 acupoint) groups (−4.935; lower limit = −15.757, upper limit = 5.887; *P* = 0.371). For the acupressure analysis, there was a significant difference in the mean VAS score after intervention between the SP6 acupoint and control (rest/light touch at SP6/nonacupoint acupressure) groups, favoring the SP6 acupoint group (−1.011; lower limit = −1.622, upper limit = −0.400; *P* = 0.001). Sensitivity analyses demonstrated good reliability of the meta-analyses findings. These findings suggest that acupuncture at SP6 is not more effective than acupuncture at an unrelated acupoint in the relief from primary dysmenorrhea. Acupressure at SP6 may be effective in the relief from primary dysmenorrhea. High-quality randomized controlled trials are needed to confirm these findings.

## 1. Introduction

Primary dysmenorrhea is a common gynecological condition, particularly in adolescents, that is characterized by cramping in the lower abdomen during menstruation [1]. Other symptoms may include nausea, vomiting, diarrhea, fatigue, fever, headache, and lightheadedness [2]. Reports on the incidence of primary dysmenorrhea vary considerably, ranging from 20% to up to 90% of menstruating females [1]. Approximately, 10% of women who experience primary dysmenorrhea suffer severe symptoms [3], which can disrupt activities of daily living, increase absenteeism, and reduce quality of life [2, 4–7]. 

Treatment for primary dysmenorrhea includes a variety of pharmacological, nonpharmacological, and surgical options. Surgery, including uterine nerve ablation and hysterectomy, is typically reserved for severe refractory cases of primary dysmenorrhea [1]. Common pharmacological interventions include nonsteroidal anti-inflammatory drugs (NSAIDs) and oral contraceptives [1, 4]. NSAIDs are most frequently used and are effective in the relief from symptoms in many patients with primary dysmenorrhea [1, 4]. Although NSAIDs and other pharmacological treatments for dysmenorrhea generally provide pain relief, the use of these treatments can be costly and associated with adverse events [2, 8]. Hence, there is a need for effective nonpharmacological treatment options for this condition.

Nonpharmacological treatments for primary dysmenorrhea include bed rest, exercise, application of heat packs, and alternative treatments such as acupuncture and acupressure [4, 9–11]. Of these treatments, acupuncture and acupressure have been widely investigated. Indeed, a number of systematic reviews published within the last decade have examined the use of acupuncture and/or acupressure for the treatment of dysmenorrhea. These reviews, however, have either included studies that involved the use of various acupoints [10, 11], multiple conditions (i.e., not just dysmenorrhea) [12], or moxibustion in addition to acupuncture/acupressure [13, 14]. To our knowledge, there has been no comprehensive review of acupuncture/acupressure studies involving the application of either of these approaches alone to a single, common acupoint for the treatment of primary dysmenorrhea.

The Sanyinjiao (SP6) acupoint, located medially four-finger wide above the ankle, is commonly used for both acupuncture and acupressure, and is thought to offer relief from gynecologic disorders, including dysmenorrhea [15, 16]. In recent years, a number of studies have evaluated the efficacy of acupuncture or acupressure at the SP6 acupoint for relieving pain associated with primary dysmenorrhea [16–24]. Many of these studies have included a relatively small number of participants and the findings have been somewhat inconsistent. Hence, we carried out a meta-analysis to evaluate the effectiveness of acupuncture or acupressure at the SP6 acupoint in relieving pain associated with primary dysmenorrhea. 

## 2. Materials and Methods

### 2.1. Search Strategy

For acupuncture, we searched PubMed, the Cochrane Library, Google Scholar, and Current Controlled Trials databases (up to December 17, 2012) using combinations of the terms menstrual, dysmenorrhea, acupuncture, Sanyinjiao, and SP6. For acupressure, the above databases were searched (up to December 17, 2012) using combinations of the terms menstrual, dysmenorrhea, acupressure, Sanyinjiao, and SP6. EMBASE was not searched due to the lack of access.

### 2.2. Selection Criteria

Randomized controlled trials in which acupuncture or acupressure specifically at the SP6 acupoint were used for the treatment of primary dysmenorrhea with pain intensity as a measured outcome were eligible for inclusion in the meta-analysis. Trials involving simultaneous acupuncture or acupressure to combinations of acupoints were excluded. Trials involving acupuncture-like transcutaneous electrical nerve stimulation, moxibustion, and other acupuncture-related techniques (except acupressure) were also excluded. Only English-language articles were eligible for inclusion.

### 2.3. Data Extraction and Quality Assessment

Searches were performed and data extracted by two independent reviewers. Each trial identified in the search was evaluated for design, patient eligibility criteria, and outcome measures. Any disagreement between reviewers concerning the eligibility of a trial was resolved by consulting with a third review. Duplicate records were excluded based on review of titles. The abstracts of all remaining articles were reviewed and any duplicate data sets were excluded. All remaining articles were reviewed in full.

Quality assessment of the trials included in the meta-analyses was performed by each reviewer according to previously described criteria [25]. 

### 2.4. Outcome Measure

The outcome measure of interest was the effect of treatment (acupuncture or acupressure) on pain as measured using the visual analogue scale (VAS), for which a score of 0 indicates no pain and a score of 10 indicates the worst pain imaginable.

### 2.5. Statistical Analyses

Mean and standard deviations were calculated for VAS scores and were compared among participants who were treated with acupuncture/acupressure and control. A *χ* ^2^-based test of homogeneity was performed and the inconsistency index (*I* ^2^) statistic was determined. If *I* ^2^ was >50% or >75%, the trials were considered to be heterogeneous or highly heterogeneous, respectively. If *I* ^2^ was <25%, the studies were considered to be homogeneous. If the *I* ^2^ statistic (>50%) that indicated heterogeneity existed between studies, a random-effects model was calculated. Otherwise, fixed-effects models were calculated. Pooled summary statistics of the difference in the mean for the individual studies are shown. Pooled differences in means were calculated and a two-sided *P* value < 0.05 was considered to indicate statistical significance. Moreover, sensitivity analysis was performed based on the leave-one-out approach. All analyses were performed using Comprehensive Meta-Analysis statistical software, version 2.0 (Biostat, Englewood, NJ, USA).

## 3. Results

### 3.1. Selection of Trials

#### 3.1.1. Acupuncture

**Figure 1:**
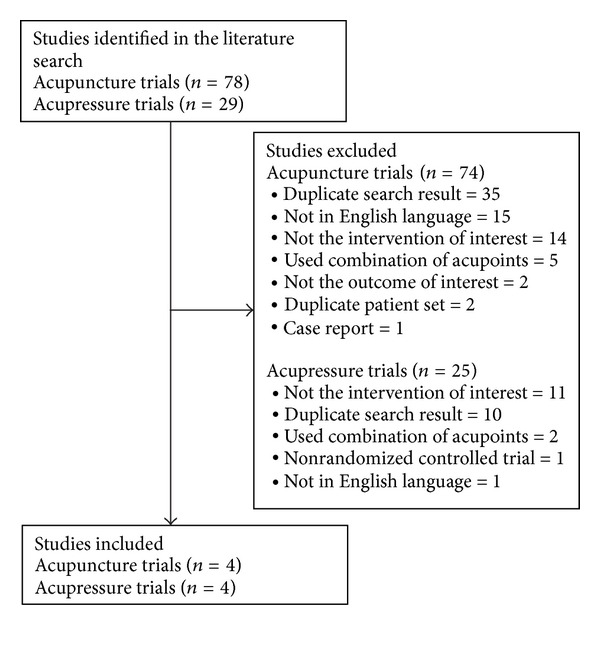
Flow diagram of trial selection.

A total of 78 trials were identified in the literature search (Figure 1). Of these, 74 were subsequently excluded after abstract review for the following reasons: duplicate search results (*n* = 35), not in English language (*n* = 15), not the intervention of interest (*n* = 14), used a combination of acupoints (*n* = 5), not the outcome of interest (*n* = 2), duplicate patient set (*n* = 2), and case report (*n* = 1). Hence, a total of four trials met the inclusion criteria [17–20].

#### 3.1.2. Acupressure

A total of 29 trials were identified in the literature search (Figure 1). Of these, 25 were subsequently excluded after abstract review for the following reasons: not the intervention of interest (*n* = 11), used combination of acupoints (*n* = 2), nonrandomized controlled trial (*n* = 1), and not in English language (*n* = 1). Hence, a total of four trials met the inclusion criteria [21–24].

### 3.2. Trial Characteristics

#### 3.2.1. Acupuncture

The characteristics of the acupuncture trials are summarized in Table 1. The trials included a total of 358 participants (range: 40 to 200 participants) who received either electroacupuncture (*n* = 3 trials) or manual acupuncture (*n* = 1) at the SP6 acupoint as a treatment for primary dysmenorrhea. Participants in all but one trial [17] were diagnosed with primary dysmenorrhea according to the Primary Dysmenorrhea Consensus Guideline [26]. Liu et al. did not specify how participants were diagnosed [17]. Each trial included at least one control group of participants who received acupuncture at the GB39 acupoint. Three trials also included a control group of participants who received acupuncture at a nonacupoint [17–19], and three trials included an additional control group of participants who did not receive acupuncture [17–19]. Acupuncture was applied from 5 to 30 min during menstruation and pain was assessed before and after (up to 30 minutes) treatment. Pain was also assessed during acupuncture in one study [17]. Pain was assessed using VAS in three of the four trials [17–19], whereas dysmenorrhea score criteria were used in one trial [20].

#### 3.2.2. Acupressure

The characteristics of the acupressure trials are summarized in Table 2. The trials included a total of 231 participants (range: 30 to 86 participants) who received acupressure at the SP6 acupoint as a treatment for primary dysmenorrhea. None of the reports specified details about the diagnosis of dysmenorrhea. The control groups varied between trials and included light touch at the SP6 acupoint in one trial [21], acupressure at a nonacupoint in another trial [22], and rest (i.e., no physical intervention) in the other two trials [23, 24]. In all trials, acupressure was initially applied by researchers for 20 to 30 minutes during menstruation. In two trials, acupressure was subsequently applied for 20 min by participants during menstruation [23, 24]. Pain was assessed before and immediately after acupressure in all trials, 30 min and 1, 2, and 3 h after treatment in two trials [21, 22], and after 3 months of ongoing treatment in two trials [23, 24]. Pain was assessed using the VAS in all four trials. The VAS for anxiety, the Short-Form McGill Pain Questionnaire (SF-MPQ), and the Short-Form Menstrual Distress Questionnaire (SF-MDQ) were also used in two trials [23, 24].

### 3.3. Effect of Treatment of Pain

#### 3.3.1. Acupuncture

The key outcomes from each individual trial are summarized in Table 1. Of the trials comparing pain with acupuncture at the SP6 versus GB39 acupoint, one reported that the decrease in VAS pain was significantly more pronounced in the SP6 group compared with the GB39 group [18]. In one of the other trials, pain, assessed using dysmenorrhea score criteria, was significantly decreased after acupuncture at the SP6 acupoint, but not the GB39 acupoint [20]. All three trials comparing acupuncture at the SP6 acupoint versus no acupuncture reported that the decrease in VAS pain was significantly more pronounced with acupuncture at the SP6 acupoint [17–19]. Of the three trials comparing acupuncture at the SP6 acupoint with acupuncture at a nonacupoint, one reported that the decrease in VAS pain was significantly more pronounced with acupuncture at the SP6 acupoint [18] and two found no difference between the groups [17, 19]. 

**Figure 2:**
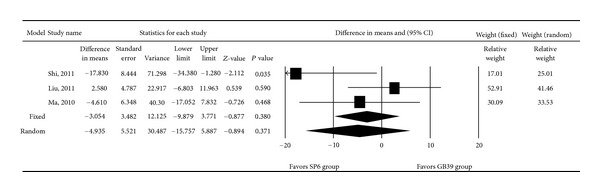
Forest plot showing differences in mean using the visual analogue scale pain score for trials in which women with dysmenorrhea received acupuncture at the SP6 or GB39 acupoint. Data are presented as the difference in means with the 95% confidence interval (CI). *P* < 0.05 indicates a statistically significant difference.

One of the trials identified in the search [20] did not report mean VAS scores before and after treatment and could not be included in the meta-analysis examining the effect of acupuncture on dysmenorrhea-associated pain. Hence, only three trials were included in the meta-analysis [17–19]. There was heterogeneity in the mean VAS score reduction among the three studies (*Q* = 4.507, *I* ^2^ = 55.63%, and *P* = 0.105); therefore, a random-effects model of analysis was used. Pooled differences in mean VAS score reductions revealed that there was no significant difference between the SP6 and GB39 groups (*P* = 0.371, Figure 2). The pooled differences in mean VAS score reduction ranged from −15.757 to 5.887; the overall mean difference was −4.935.

#### 3.3.2. Acupressure

The key outcomes from each individual trial are summarized in Table 2. All trials found that pain scores (VAS, SF-MPQ, or SF-MPQ) were significantly lower in the acupressure group compared with the control group after intervention. In one trial, VAS anxiety scores were also found to be significantly lower in the acupressure group compared with the control group after intervention [24].

**Figure 3:**
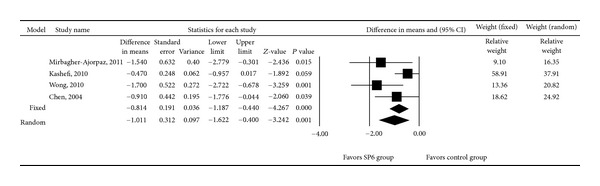
Forest plot showing differences in mean using the visual analogue scale pain score for trials in which women with dysmenorrhea received acupressure at the SP6 acupoint or control treatment. Data are presented as the difference in means with the 95% confidence interval (CI).*P* < 0.05 indicates a statistically significant difference.

All four acupressure trials were included in the meta-analysis examining the effect of acupressure on dysmenorrhea-associated pain. There was heterogeneity in the mean VAS score after intervention among the four studies (*Q* = 6.169, *I* ^2^ = 51.37%, and *P* = 0.104); therefore, a random-effects model of analysis was used. Pooled differences in mean VAS scores after intervention revealed a significant difference between the SP6 and control groups (*P* = 0.001, Figure 3). The pooled differences in the mean VAS score after intervention ranged from −1.622 to −0.400, with the pooled mean difference being −1.011.

### 3.4. Quality Assessment and Sensitivity Analysis

#### 3.4.1. Acupuncture

Overall, the acupuncture trials identified had adequate sequence generation, allocation concealment, and addressed incomplete outcome data (Table 3). We were only able to determine that one of the acupuncture trials identified [17] was free of selective reporting. We were not able to confirm that any of the trials were free of other biases.

#### 3.4.2. Acupressure

Overall, the acupressure trials identified had a high associated risk of bias due to sequence generation, allocation concealment, and blinding (Table 3). Incomplete outcome data were adequately addressed in all trials. We were unable to determine if any of the acupressure trials identified were free of selective reporting or other bias.

**Figure 4:**
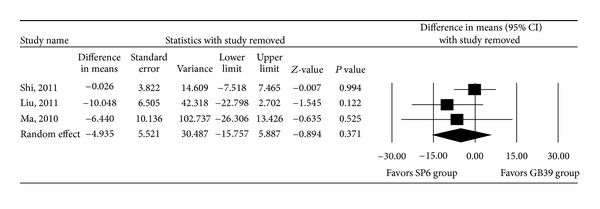
Sensitivity analysis for the influence of individual studies on the pooled estimate (as determined using the leave-one-out approach) of the visual analogue scale pain score for trials in which women with dysmenorrhea received acupuncture at the SP6 or GB39 acupoint. Data are presented as the difference in means with the 95% confidence interval (CI). *P* < 0.05 indicates a statistically significant difference.

**Figure 5:**
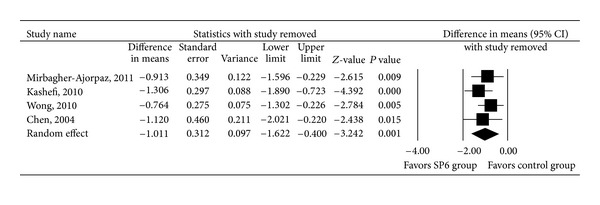
Sensitivity analysis for the influence of individual studies on the pooled estimate (as determined using the leave-one-out approach) of the visual analogue scale pain score for trials in which women with dysmenorrhea received acupressure at the SP6 acupoint or control treatment. Data are presented as the difference in means with the 95% confidence interval (CI). *P* < 0.05 indicates a statistically significant difference.

Figures 4 and 5 show the results of meta-analysis with one study removed in turns. These results demonstrate that even when each trial was excluded from the meta-analysis, the direction and magnitude of the pooled estimates did not vary markedly. Moreover, across all meta-analyses, we found that random-effects models provided similar estimates to those of fixed models. These findings are indicative of good meta-analyses reliability.

## 4. Discussion

This is the first systematic review to evaluate the effectiveness of acupuncture or acupressure at the SP6 acupoint for relieving pain associated with primary dysmenorrhea. Our findings suggest that acupuncture at the SP6 acupoint may not be more effective than acupuncture at an unrelated (GB39) acupoint for the relief of dysmenorrhea-associated pain (assessed using a VAS). In contrast, our findings suggest that acupressure at the SP6 acupoint may provide more effective relief from dysmenorrhea-associated pain than control interventions.

Findings among the acupuncture trials identified in our literature search were generally inconsistent. The only consistent finding was that acupuncture at the SP6 acupoint resulted in a greater decrease in VAS pain than no acupuncture [17–19]. Other differences between treatment groups (SP6 versus GB39, SP6 versus nonacupoint acupressure) were apparent in some studies, but not others. This lack of consistency is reflected in our meta-analysis finding that acupuncture at the SP6 acupoint did not provide better pain relief from primary dysmenorrhea than did acupuncture at the GB39 acupoint.

There was marked heterogeneity among the acupuncture trials that may have affected our meta-analysis findings and explain the between-trial disparity. Notably, the timing and length of acupuncture differed between some studies (see Table 1). Further, participants in three trials [17–19] were instructed/permitted to use aspirin for pain relief during the trial. Use of aspirin may have confounded any acupuncture-related pain relief. 

The acupressure trials identified in our literature review consistently reported that acupressure at the SP6 acupoint resulted in better pain relief than that of control treatment, the nature of which varied between studies. Unsurprisingly, our meta-analysis also revealed that acupressure at the SP6 acupoint resulted in significantly better pain relief. 

As with the acupuncture trials, there was distinct heterogeneity among the acupressure trials. There were between-trial differences in the timing and application of acupressure, and, as already highlighted, a lack of consistency in the types of control interventions used. Further, participants in some trials were allowed to take pain medication before acupressure; however, the timing of allowed medication differed, ranging between >3 hours before treatment [21], >4 hours before treatment [24], and >6 hours before treatment [23]. Another difference between studies that may have affected the pain outcome is variable participant positioning during acupressure, that is, prone [22, 24], supine [21], or seated cross-legged [23]. Finally, none of the reports described how primary dysmenorrhea was diagnosed; hence, it is possible that some of the participants in the trials may have had secondary dysmenorrhea. 

Our meta-analyses have several limitations that must be acknowledged. Firstly, and perhaps most notably, only a small number of trials met the criteria for inclusion, thus reducing the power of our analyses. Secondly, we only searched the English-language literature. It is possible that other relevant trials may have been identified if we had searched the literature in other languages. Finally, and as already discussed, there was clear heterogeneity among the studies identified, which may have affected the outcomes of the meta-analyses.

## 5. Conclusions

In conclusion, there is insufficient high-quality evidence available in the current literature regarding the effectiveness of acupuncture or acupressure at the SP6 acupoint for the treatment of pain associated with primary dysmenorrhea. Hence, the findings from our meta-analyses are by no means definitive. Nevertheless, our findings do suggest that acupuncture at the SP6 acupoint may not be more effective in relieving pain than acupuncture at an unrelated acupoint. Further, our findings suggest that acupressure at the SP6 acupoint may provide more effective pain relief than that of control treatment. Clearly, there is a need for high-quality, randomized controlled trials to clarify the effectiveness of acupuncture/acupressure at the SP6 acupoint for the treatment of pain resulting from primary dysmenorrhea.

## Figures and Tables

**Table 1:** Characteristics of acupuncture trials identified in the literature search.

First author, year (location)	Number of participants	Intervention	Control	Assessment	Outcomes
Shi, 2011 [19](China)	40	Electroacupuncture at SP6 for 30 min	Electroacupuncture at unrelated acupoint (GB39), electroacupuncture at nonacupoint, and no electroacupuncture	Pain assessed by VAS before and after intervention	There were significant differences in VAS scores between the SP6 and no acupuncture groups after intervention (24.7 versus 48.2, *P* < 0.05). There were no significant differences in VAS scores between the 3 acupuncture groups

Liu, 2011 [17] (China)	200	Electroacupuncture at SP6 for 30 min for 2 menstrual cycles	Electroacupuncture at unrelated acupoint (GB39), electroacupuncture at nonacupoint, and no electroacupuncture	Pain assessed by VAS before intervention, 5, 10, 30 min during intervention, and 30 min after intervention	The mean decrease in VAS score was significantly greater in all acupuncture groups compared with the no acupuncture group (SP6: −15.56, *P* < 0.05; GB39: −18.14, *P* < 0.05; nonacupoint: −10.96, *P* < 0.05) There were no significant differences in VAS scores between the 3 acupuncture groups

Ma, 2010 [18] (China)	52	Electroacupuncture at SP6 for 10 min on day 1, 30 min on days 2 and 3	Electroacupuncture at unrelated acupoint (GB39), electroacupuncture at nonacupoint, and no electroacupuncture	Pain assessed by VAS before intervention, 5, 10, and 30 min after intervention	There were significantly greater reductions in VAS scores in the SP6 group compared with the other groups at each time after intervention (all *P* < 0.05)

Yu, 2010 [20] (China)	66	Manual acupuncture at SP6 for 5 min	Manual acupuncture at unrelated acupoint (GB39)	Pain assessed before and after intervention according to dysmenorrhea score criteria	The pain score significantly decreased after intervention in the SP6 group (before = 11.20 versus after = 8.17, *P* < 0.05), but not in the GB39 group

VAS: visual analogue scale.

**Table 2:** Characteristics of acupressure trials identified in the literature search.

First author, year (location)	Number of participants	Intervention	Control	Assessment	Outcomes
Mirbagher-Ajorpaz, 2011 [21] (Iran)	30	Acupressure at SP6 for 20 min applied by researcher	Light touch at SP6 for 20 min applied by researcher	Dysmenorrhea severity measured using VAS before and immediately, 30 min, and 1, 2, and 3 h after treatment	There were significant differences in VAS scores between the acupressure and control groups immediately, 1, 2, and 3 h after intervention (3.50 versus 5.06, 3.30 versus 4.86, 2.40 versus 5.00, and 1.66 versus 4.80, resp., all *P* < 0.05)

Kashefi, 2010 [22] (Iran)	86	Acupressure at SP6 for 30 min applied by researcher during the first 24 h of menstrual cycle for 2 cycles	Acupressure at a nonacupoint 30 min applied by the researcher during the first 24 h of menstrual cycle for 2 cycles	Dysmenorrhea severity assessed by VAS before and immediately, 30 min, and 1, 2, and 3 h after intervention	For the first cycle, there were significant differences in VAS scores between the acupressure and control groups 30 min, 1, 2, and 3 h after intervention (4.90 versus 6.06, 4.38 versus 6.23, 4.55 versus 6.34, and 5.34 versus 6.81, resp., all *P* < 0.05). For the second cycle, there were significant differences in VAS scores between the acupressure and control groups immediately, 30 min, 1, 2, and 3 h after intervention (5 versus 6.16, 4.86 versus 6.04, 4.72 versus 6.04, 4.72 versus 6.04, 4.60 versus 6.58, and 5.67 versus 7.06, resp., all *P* < 0.05)

Wong, 2010 [23] (China)	46	Acupressure at SP6 for 20 min applied by researcher at initial intervention 20 min acupressure self-treatment upon waking and at bedtime during the first 3 days of the next 3 menstrual cycles	Rest for 20 min at initial intervention 20 min rest upon waking and at bedtime during the first 3 days of the next 3 menstrual cycles	Dysmenorrhea severity assessed immediately after first treatment and after 3 months using VAS, SF-MPQ, and SF-MDQ	There were significant differences in VAS scores and SF-MPQ between the acupressure and control groups immediately after initial intervention (VAS: 4.11 versus 5.81, *P* < 0.05; SF-MPQ: 5.26 versus 7.38, *P* < 0.05) There were significant differences in VAS scores of SF-MPQ and SF-MDQ between the acupressure and control groups after 3 months of self-care (VAS: 2.79 versus 4.30, *P* < 0.05; SF-MPQ: 3.53 versus 5.81, *P* < 0.05; SF-MDQ: 23.96 versus 26.61, *P* < 0.05)

Chen, 2004 [24] (Taiwan)	69	Acupressure at SP6 for 20 min applied by researcher at initial intervention 20 min acupressure self-treatment during next menstrual cycle	Rest for 20 min at initial intervention 20 min rest during next menstrual cycle	Dysmenorrhea severity assessed using VAS for pain and VAS for anxiety	There were differences in VAS pain and anxiety scores between the acupressure and control groups after the initial intervention (pain: 3.88 versus 4.79; anxiety: 3.13 versus 3.74) There were differences in VAS pain scores between the acupressure and control groups after self-treatment (2.92 versus 3.04)

VAS: visual analogue scale; SF-MPQ: Short-Form McGill Pain Questionnaire; SF-MDQ: Short-Form Menstrual Distress Questionnaire.

**Table 3:** Quality assessment of trials identified in the literature search: risk of  bias.

	Sequence generation adequate	Allocation concealment adequate	Blinding adequate	Incomplete outcome data addressed	Free of selective reporting	Free of other bias
Acupuncture trials						
Shi et al., 2011 [19]	+	+	+	+	?	?
Liu et al., 2011 [17]	+	+	+	+	+	?
Ma et al., 2010 [18]	+	+	+	+	?	?
Yu et al., 2010 [20]	+	+	−	+	?	?
Acupressure trials						
Mirbagher-Ajorpaz et al., 2011 [21]	+	−	−	+	?	?
Kashefi et al., 2010 [22]	−	−	+	+	?	?
Wong et al., 2010 [23]	−	−	−	+	?	?
H. M. Chen and C. H. Chen, 2004 [24]	−	−	−	+	?	?

+: low risk of  bias; −: high risk of  bias; ?: unclear risk of  bias.
